# Hydrogen-rich saline promotes microglia M2 polarization and complement-mediated synapse loss to restore behavioral deficits following hypoxia-ischemic in neonatal mice via AMPK activation

**DOI:** 10.1186/s12974-019-1488-2

**Published:** 2019-05-18

**Authors:** Xili Chu, Lili Cao, Zhuoya Yu, Danqing Xin, Tingting Li, Weiwei Ma, Xin Zhou, Wenqiang Chen, Dexiang Liu, Zhen Wang

**Affiliations:** 1 Department of Physiology, Shandong University School of Basic Medical Sciences, 44 Wenhua Xi Road, Jinan, Shandong 250012 People’s Republic of China; 2 Department of Medical Psychology, Shandong University School of Basic Medical Sciences, 44 Wenhua Xi Road, Jinan, Shandong 250012 People’s Republic of China; 3 Shandong University Qilu Hospital, Jinan, Shandong People’s Republic of China

**Keywords:** M2 polarization, Complement, Synapse loss, Hydrogen-rich saline, Hypoxia-ischemia

## Abstract

**Background:**

Hypoxia-ischemia (HI) during the perinatal period is one of the most common causes of acute mortality and chronic neurologic morbidity. Hydrogen-rich saline (HS) treatment in neonatal mice has been reported to alleviate brain injury following HI, but the mechanisms involved are not known.

**Methods:**

A modified version of the Rice-Vannucci method for the induction of neonatal HI brain injury was performed on postnatal day 7 mouse pups. Animals or BV2-cells received HS and an AMPK inhibitor at indicative time post-injury.

**Results:**

In the current study, we show that HS treatment attenuated the accumulation of CD11b^+^/CD45^high^ cells, suppressed HI-induced neuro-inflammation, induced microglial anti-inflammatory M2 polarization, was associated with promoting AMPK activation, and inhibited nuclear factor-κB activation as demonstrated both in vivo and in vitro. In addition, HS treatment reversed HI-induced neurological disabilities, was associated with improving damaged synapses, and restored the expression levels of synaptophysin and postsynaptic density protein 95 following HI insult. Furthermore, HI insult which increased levels of complement component C1q, C3, and C3aR1 was observed. Importantly, C1q deposited in the infarct core and lesion boundary zone following HI injury, was found to co-localize within regions of synapse loss, whereas HS treatment reversed these effects of HI on synapse loss and complement component levels. Notably, the AMPK inhibitor reversed the beneficial effects of HS as described above.

**Conclusions:**

These results demonstrate that HS restored behavioral deficits after HI in neonatal mice. These beneficial effects, in part, involve promoting microglia M2 polarization and complement-mediated synapse loss via AMPK activation.

**Electronic supplementary material:**

The online version of this article (10.1186/s12974-019-1488-2) contains supplementary material, which is available to authorized users.

## Introduction

Neonatal hypoxic-ischemic encephalopathy (HIE) is one of the most common causes of morbidity and mortality in infants. Regardless of significant progress in elucidating the mechanisms underlying HIE, there remains no well-established clinically effective treatments to reduce brain damage and its long-term sequelae in these children[1]. Therefore, exploring new therapeutic interventions and targets to minimize such neurological consequences are urgently needed.

Following HI an immediate activation of microglia occurs, which is involved with mediating the brain injury observed [2, 3]. Activated microglia significantly contribute to neuro-inflammation and represent the predominant source for inflammatory mediators in the central nervous system (CNS). Microglia can be polarized into two extreme states: classical (M1) and alternative (M2) phenotypes. M1-activated microglia express CD16 and CD86 and release inflammatory cytokines, including tumor necrosis factor (TNF)-a and interleukin (IL)-1β, which enhance brain damage. M2 -activated microglia express CD206 and YM-1 and release anti-inflammatory cytokines and growth factors, including IL-10 and transforming growth factor β (TGF-β), which leads to neuroprotection. Findings from a number of studies have indicated that mediating microglial phenotype shifts associated with CNS damage, including HI in neonatal animals can exert beneficial effects with regard to neurological recovery [4, 5]. Accordingly, by focusing on M1/M2 switching processes following HI, a promising therapeutic target for improving stroke outcomes can be achieved [6].

Hydrogen gas represents a relatively new medical gas that exerts organ-protective effects through regulating oxidative stress, inflammation, and apoptosis [7–9]. Several lines of evidence demonstrate that hydrogen offers beneficial effects in various neurological diseases, such as Alzheimer’s disease, Parkinson’s disease, cerebral ischemia, and spinal cord injury [7, 10]. Hydrogen also affords neuroprotection against brain injury in a neonatal model of hypoxia-ischemic (HI) [11, 12]. Within our laboratory, we have found that hydrogen-rich saline (HS) exerts neuroprotection against HI insult in neonatal mice by mediating endoplasmic reticulum stress and autophagic machinery [13]. However, relatively little is known regarding the potential for HS to restore HI-induced behavioral deficits and whether such effects might be involved with anti-inflammatory action and mediation of synapse remodeling.

## Materials and methods

### Materials

Compound C, LPS, 2,3,5-triphenyltetrazolium chloride monohydrate (TTC), and Cresyl violet acetate were purchased from Sigma-Aldrich (St Louis, MO, USA). Rabbit anti-Synaptosin (Syn), PSD95, p-NF-κB, NF-κB, p-AMPKα, AMPKα, and p-IkBα antibodies were from Cell Signaling (Beverly, MA, USA). Rabbit anti-CD16 and CD206 antibodies were from Abcam (Cambridge, UK). Mouse anti-C1q antibodies were from Abcam. APC anti-mouse CD45 and FITC anti-mouse/human CD11b were from Biolegend (San Diego, CA, USA). Details regarding antibodies used for Western blotting and flow cytometric analysis are listed in Additional file 1: Table S1.

### Mouse HI model and treatment

Pregnant C57BL/6J mice (> 15 days gestation) were purchased from the animal center of Shandong University and were maintained under standardized environmental conditions where they gave birth. The HI animal model utilized in this experiment was based on that of the Rice–Vannucci model, with minor modifications. In previous reports, the right common carotid artery of mice was isolated and ligated. After a 30-min period of recovery at room temperature, the pups were exposed to hypoxia injury for 120 min in an anoxic chamber (humidified 8% O2 + 92% N2). However, when we used this method to build the model, about 40% of the mice died. And then we modified the method. In brief, on postnatal day 7 (P7), mice of both sexes were anesthetized with 2.5% isoflurane on day 7 after birth, and following a skin incision, the right common carotid artery was isolated and ligated. After a 60-min period of recovery at room temperature, the pups were exposed to hypoxia injury for 90 min in an anoxic chamber (humidified 8% O2 + 92% N2). After many preliminary experiments, the modified method can successfully establish the model and reduce the mouse mortality. Sham controls were anesthetized with 2.5% isoflurane on day 7 after birth, and the right common carotid artery was separated but not ligated.

The mouse pups were randomly allocated into four groups: (1) Sham + vehicle (saline), (2) HI + vehicle (saline), (3) HI + HS, and (4) HI + HS + Compound C. HS was prepared in 100-ml bottles using a hydrogen-generator and dissolved in physiological saline under 0.4 MPa pressure at 4 °C for 12 h. HS was freshly prepared every time before the experiment to ensure a hydrogen concentration greater than 0.6 mM. The content of hydrogen was confirmed using a dissolved hydrogen portable meter. HS-saturated saline was administered by peritoneal injection (i.p. 5 ml/kg). HS was administered at 1, 2, and 3 days after HI insult. Compound C (an AMPK inhibitor) was initially dissolved in DMSO and then diluted in saline, with the concentration of DMSO being less than 1% of the total volume. In group 4, Compound C was injected intraperitoneally and followed 30 mins later by HS. HI and Sham groups were injected with an equal amount of vehicle (PBS) as based upon their body weights. Administration route and dose of HS was according to that used in our previous studies [13]. The administration route and dose of Compound C were according to that of McCullough et al [14].

The experimental schedule of tissue analyses for the first experiment is presented in Additional file 2: Figure S1A, and the behavior experimental schedule for the second experiment is presented in Additional file 2: Figure S1B.

### Measurement of infarct size

Brains of mouse pups (*N* = 4/group) were rapidly removed at 3 days post-HI and hardened in a refrigerator (− 20 °C) for approximately 20 min for slicing. Each brain was cut into 4 coronal slices of 1-mm thickness and counterstained using 2% TTC solution for 30 min at 37 °C in a warm box. Infract volume quantification was then determined using procedures described previously [13].

### Collection of tissue and Immunohistochemistry and Immunofluorescence preparation

At 3 days after HI, anesthetized mice (*N* = 4–6/group) were perfused with 4% paraformaldehyde (pH 7.4). The brain was removed and maintained in the same fixative for one day at 4 °C. Coronal sections with a thickness of 4 μm were sliced from an area ranging between − 1.60 and − 2.00 mm from bregma and stained for immunofluorescence and immunohistochemistry assays.

The immunofluorescence assay was performed as described previously [14]. In brief, brains slices were treated with the following primary antibodies: PSD95 (1:100), C1q (1:100), Iba-1 (1:100), CD16 (1:100), or CD206 (1:100). The Magna Fire SP system and fluorescent microscopy (OLYMPUS-BX51) were utilized for microphotographic analyses. The number of PSD95-positive cells in 6 microscopic fields randomly selected from the infarct’s core region of cortex and lesion boundary zone were determined (× 200 magnification). For CD16/Iba-1 and/or CD206/Iba-1, the number of double-positive cells in 6 microscopic fields randomly selected from the infarct’s core region of cortex was counted (× 200 magnification). The total number of positive cells within each section was then expressed as the average value of the 6 images per section.

For immunohistochemistry staining each brain slice was heated to 60 °C for 90 min. The slice was then dewaxed, dehydrated, and treated with antibodies according to standard procedures. After blocking of endogenous peroxidase, each slice was incubated at 4 °C overnight with C1q (1:100) or Iba-1 (1:100), followed by secondary antibodies for 30 min at room temperature. Antibody binding analysis was performed with use of a DAB kit, and each slide was evaluated by microscopy with use of the abovementioned Magna Fire SP system. C1q^+^ cell numbers within the infarct’s core region of the cortex and in the lesion boundary zone (*N* = 6 mice/group) were determined within 3 microscopic fields (× 200 magnification). The number of C1q^+^ and Iba-1^+^ cells within each slice was then averaged from values obtained within these 3 images. This calculated value was then expressed as the percent of C1q^+^ and Iba-1^+^ cells relative to those obtained within the Sham group.

### BV-2 cell culture

BV-2 cells were obtained from American Type Culture Collection. BV-2 cells (*N* = 4/group) seeded onto 6-well plates were incubated overnight and treated with/without HS or with/without LPS (500 ng/ ng/mL) utilizing DMEM:F12 media. Cells were then collected at the times indicated for use in future experiments.

### Microglia/Macrophage isolation from brain and analysis using flow cytometry

Single cells were prepared as described previously [12]. Briefly, at 3 days after HI insult, the right cerebral cortex (*N* = 4/group) tissue from each group was placed in a pre-cooled Hank balanced salt solution. The tissue was cut into approximately 1-mm^3^ blocks, digested with 10 ml digestive solution at 37 °C for 40 min, compressed, and a cell suspension was generated. The cell suspension was filtered through a 70-μm sieve, centrifuged at 420×*g* for 10 min and the pellet was resuspended with 4 mL 40% Percoll solution (GE Healthcare BioSciences). Then, 4 mL 70% Percoll solution was slowly added to the lower cell suspension using a syringe and centrifuged at 500×*g* for 20 min. One part of the 10× PBS was combined with 9 parts of Percoll stock solution for preparation of an isotonic suspension of Percoll, which we defined as a 100% suspension of Percoll. The 100% Percoll was diluted with 1× PBS to generate the expected density of Percoll separation solution for cell isolation. Cells were harvested from the interface using different concentrations of Percoll solution and rinsed once with PBS containing 0.2% of BSA. Cells were stained with the antibodies, anti-mouse CD11b-FITC or mouse CD45-APC, for evaluation of CNS-associated phagocytes (CD11b^+^/CD45^high^ cells). Flow cytometric analysis was performed using a FACS flow cytometer C6 (BD Biosciences).

### Reverse transcription-PCR

The extracted cortex or cells (*N* = 4/group) were frozen at − 120 °C. The Ultrapure RNA Kit was used to extract total RNA according to the manufacturers’ instructions. Then, reverse transcription was performed using the Revert Aid First Strand cDNA Synthesis Kit. PCR with specific primers was used to amplify the cDNA for β-actin, cluster of differentiation 86 (CD86), IL-1β, TGF-β, YM-1, C1q, C3, and C3aR1 (Additional file 1: Table S2). Electrophoresis was used to separate reaction products with 1.3% of agarose/TAE gel containing 0.1% of GoldView (vol/vol). The reaction products were run for 30–40 min at 90 V. The GelDocXR System was used to capture the image. Image-Pro Plus 6.0 software was used for determining band intensities and each value was normalized to β-actin.

### Western blot analysis

The extracted lesioned cortex (*N* = 4/group) was frozen at – 120 °C. For immunoblots, the frozen tissue was initially weighed and cracked. After homogenization within RIPA buffer containing protease/phosphatase inhibitors and PMSF, the tissue was centrifuged at 4 °C at 13800×*g*/10 min. The resultant supernatants were treated with 5× loading buffer, and total protein concentrations were quantified with use of the BCA Protein Assay Kits. Equivalent concentrations of proteins were run on SDS-PAGE gels after being diluted with 5× loading buffer. Proteins were initially electrophoresed for 30 min at 80 V followed by 110 V for at least 1 h and then transferred to PVDF membranes at 300 mA (1 h) utilizing a one wet transfer system. Membranes were blocked for 1 h and then treated with the following primary antibodies, β-actin, p-NF-κB, NF-κB, p-p38, p38, p-IkBa, IkBa, p-AMPKa, AMPKa, Syn, or PSD95 at 4 °C overnight. PVDF membranes were incubated with secondary antibodies at RT for 1 h. ECL kit reagents (MILLIPORE, America) were then used to develop chemiluminescent signals, which were detected using the Tanon Imaging System (Tanon-4600).

### Transmission electron microscopy (TEM)

The right side of the brain (*N* = 4/group) was quickly removed, dissected into a 1-mm^3^ tissue block, and fixed in 2.5% glutaraldehyde at 4 °C for 2 h. After washing with PBS for 3–5 times, the sample was fixed in 1% osmium tetroxide for 2 h and then incubated in gradients of ethanol solutions. It was then permeated and submerged overnight in 50/50 epoxy propane. Tissue was prepared for slicing (50-nm thick) with the use of an Ultra ultrathin slicer (EM UC 7, Leica, Germany). After staining with uranyl acetate, the sections were observed and photographed with the use of Hitachi h-7500 TEM.

### Behavioral Testing

#### Short-term neurological evaluations

At P10, P12, and P14, each group was subjected to behavioral tests (*N* = 10/group) for evaluation of short-term neurological performance. The two tests included the Negative geotaxis and Front-limb suspension test (Additional file 2: Figure S1B). Each mouse was tested three times and the average of their three scores was recorded.

For the Negative geotaxis test, each mouse was placed on a slant board (30 cm long and 45° incline) with their head pointed toward the downward slope as previously described [15]. The time required for the mouse to turn its head in 30 s was recorded. For the front-limb suspension test, both forepaws of the mouse were placed on a suspended wire and the time required for the mouse to fall from the wire within 60 s was recorded as previously described [15].

#### Long-term neurological evaluations

At P35 mice (*N* = 10/group) were subjected to two behavioral tests for evaluation of their long-term neurological performance. The experimental schedule is presented in Additional file 2: Figure S1B.

##### Novel Object Recognition Test (NORT)

Mice were placed in an open field (60 × 40 × 40 cm) for 10 min/day on 3 consecutive days prior to the onset of the experiment to accustom them to the test conditions. At the start of the test, mice were placed in the open field for 10 min with two balls of the same color present within the open field chamber. The duration of contact with the balls, including the number of times the mice touched the balls with their noses or mouths, was recorded. Between trials, the equipment and objects were cleaned with 50% ethanol to reduce any olfactory cues. At 1 h after the first test, mice were tested again with a new object (with a different shape and color) replacing the familiar object presented in the first test. The amount of time spent investigating the new or old objects was recorded. A discrimination ratio was then calculated as novel/(novel + familiar time)

##### Morris water maze (MWM) test

The water maze test was conducted in black cylindrical barrels (120 cm in diameter and 60 cm in depth). Movement of the animals during tests within the barrel was recorded with use of a tracking system. The water maze was divided into four quadrants of equal areas, and a platform was placed below the horizontal plane of the water at the center of the third quadrant. During the 5 initial days of exposure to the water maze, all animals were trained to locate the platform. The animals were placed within different quadrants in the maze, and tracking software was used to record the time required for the animals to locate the platform. On the 6th day, the location of the platform was changed and the animals were allowed to swim freely in the maze for 60 s. The time required to locate the platform was recorded. The data for latencies and swimming speeds were collected with use of the Video Tracking system SMART. Morris water maze video tracking and system is divided into two parts: test instrument and analysis software. The system can measure the memory behavior index of the animal and the computer can track and monitor it automatically. The system used the color image processing algorithm to track the motion curve of mice in real time. It can analyze the path and time of the experimental animals in four quadrants, the effective rate in six periods, the orientation angle, the average motion speed, the times of passing through the virtual platform, and the residence time of the virtual platform. The system automatically records the experimental results in groups.

### DHE staining

To measure brain or neuronal ROS production, frozen coronal sections (12 μm) of the ipsilateral hemisphere or cells were stained with DHE as previously described. Briefly, coronal sections were stained with 10 μM DHE for 30 min. After being rinsed and mounted, fluorescence images were captured using fluorescent microscopy. The DHE staining results were pixilated and quantified using the Image-pro plus image analysis system. Values were expressed relative to the fluorescence signal of respective controls.

### Statistical analysis

Data were analyzed with use of the IBM SPSS software (version 20, IBM Inc.). All values presented are expressed as the mean ± standard deviation (SD). Data from the training trials in the MWM were averaged for each mouse (total data/total number of trials per day). Daily performance scores in the negative geotaxis, front-limb suspension, and MWM tests were evaluated by repeated-measures two-way analysis of variance (ANOVA) with “days” as the within-subject factor and “group” as the between-subject factor, while daily scores were compared using *t* tests. Other data were analyzed with use of the one-way ANOVA and Tukey’s test for multiple post-hoc comparisons of means. A *p* value < 0.05 was required for results to be considered statistically significant. All “Ns” in each group for histological findings refer to the number of animals.

## Results

### HS promoted AMPK phosphorylation *in vivo and in vitro*

**Fig. 1:**
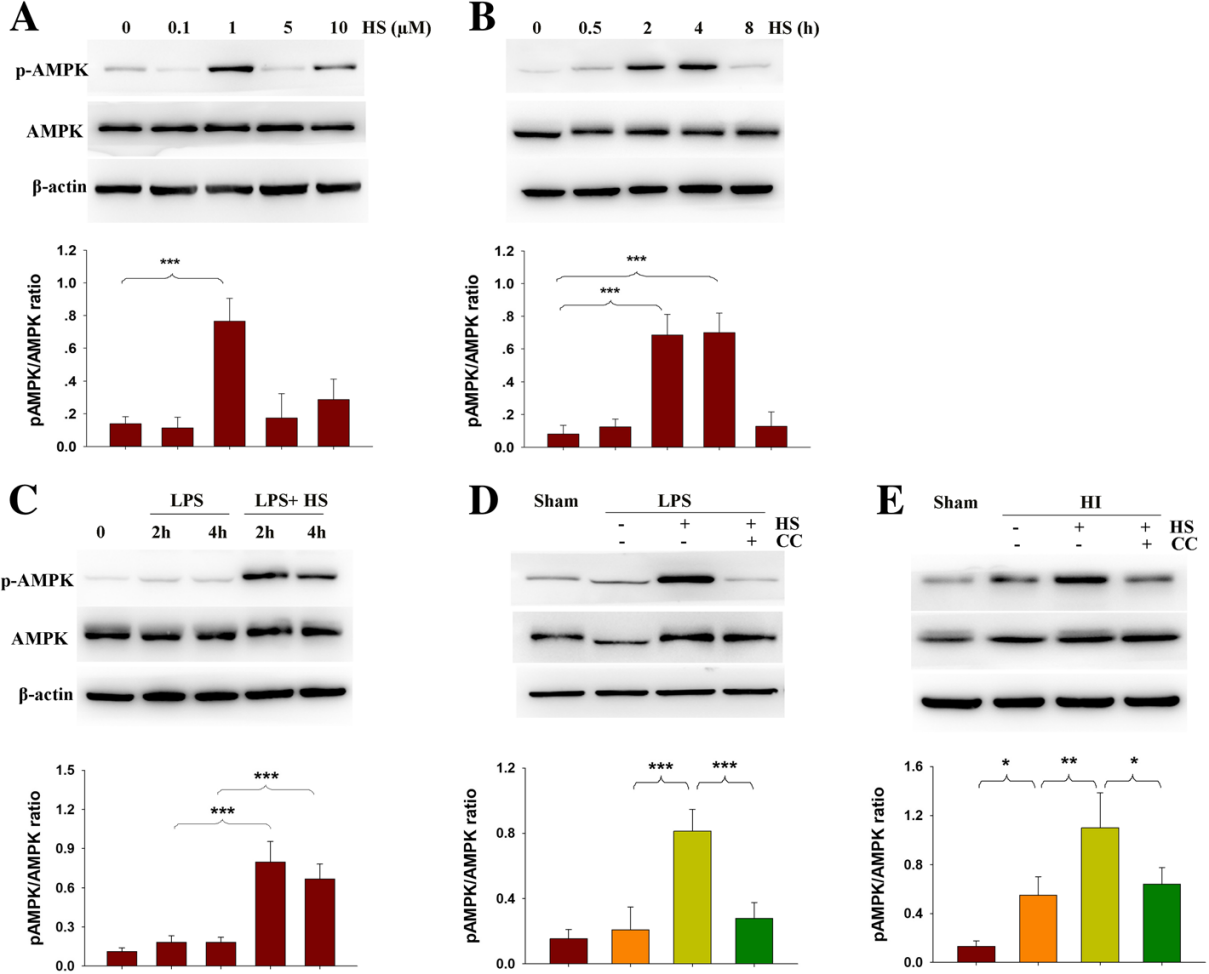
Effects of HS on AMPK activation**. a** BV-2 cells treated with different concentration of HS for 2 h. **b** BV-2 cells treated with 1 μM HS for different times. **c** BV-2 cells treated with 1 μM HS with/without 500 ng/mL LPS for 2 or 4 h. **d** BV-2 cells treated with 1 μM HS with/without 500 ng/mL LPS or 1 μM Compound C (CC) for 2 h. Levels of p-AMPK, AMPK and β-actin were determined with use of Western blot. **e** Representative immunoblots of protein levels for phosphorylated AMPK (p-AMPK), AMPK and β-actin in ipsilateral cortex at 3 days following HI insult. *N* = 4/group. Values represent the mean ± SD, *p < 0.05, **p < 0.01 according to ANOVA

In the absence of LPS, HS (1 μM) produced an overall increase in p-AMPK levels within BV-2 cells (*p* < 0.001, Fig. 1a). These effects appeared to be temporally dependent with increases observed at both 2 (*p* < 0.001) and 4 (*p* < 0.001) h after HS treatment in these BV-2 cells (Fig. 1b). LPS (500 ng/mL) significantly increased p-AMPK levels at 30 min (Additional file 3: Figure S2, *p* < 0.001) but decreased p-AMPK levels at 2 and 4 h. When LPS (500 ng/mL) was combined with 1 μM HS, the LPS-induced decrease in p-AMPK in BV-2 cells was blocked as determined at the 2- (*p* < 0.001) and 4-h (*p* < 0.001) periods following treatment (Fig. 1c). The AMPK inhibitor, Compound C (1 μM), blocked the effect of HS on AMPK activation (Fig. 1d, *p* < 0.001). As shown in Fig. 1e, p-AMPK expression within the lesioned cortex was significantly increased in the HI + Vehicle group as compared with that of the Sham group (*p* < 0.05) when determined at 3 days following HI exposure. HS treatment further upregulated p-AMPK expression (Fig. 1e, *p* < 0.01), while Compound C blocked this effect of HS on AMPK activation in the lesioned cortex (Fig. 1e, *p* < 0.05).

### HS treatment suppressed HI-induced injury and microglia activation

**Fig. 2:**
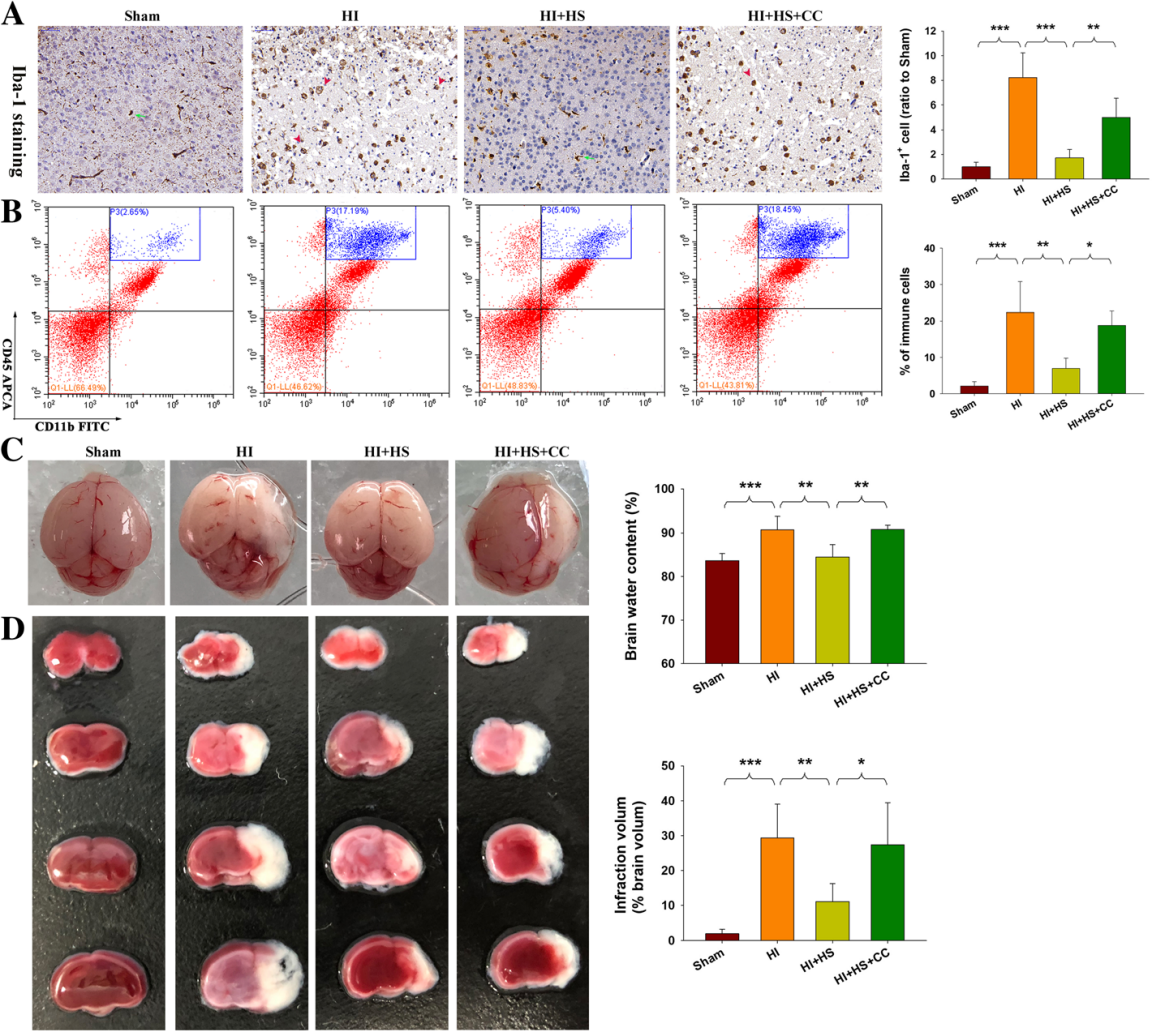
Effects of HS on HI-induced microglial activation. **a** Representative Iba-1 staining within the core of infarcted cortex at 3 days following HI insult. N = 4/group. **b** Representative flow cytometric lots of CD11b^+^/CD45^high^ cells within ipsilateral cortex at 3 days following HI insult. *N* = 4/group. Right histograms of average percentage of CNS-associated phagocytes (CD11b^+^/CD45^high^ cells) in live single immune cells from brains. **c** Representative brain images as obtained at 72 h following HI. Arrows indicate sites of significant edema. Brain water content was determined at 72 h following HI insult, *N* = 6/group. **d** Representative samples stained with TTC. Quantified infarct volume is indicated by the white area. *N* = 6/group. Values represent the mean ± SD, *p < 0.05, **p < 0.01, ***p < 0.001 according to ANOVA

Results from Iba-1 immunohistochemistry show that relatively few Iba-1 labeled cells were present in cortex of Sham mice. After HI exposure, the core region of the infarct area within the cortex displayed increased numbers of Iba-1^+^ cells, with most of the microglia exhibiting a rounded amoeboid-like appearance. HS treatment blocked this HI-induced microglial activation (*p* < 0.01, Fig. 2a). While, HI exposure remarkably increased the number of CNS-associated phagocytes (CD11b^+^/CD45^high^) within the lesioned cortex at 72 h following injury, HS treatment significantly attenuated these increased CNS-associated phagocytes (*p* < 0.001, Fig. 2b). Pre-treatment with the AMPK inhibitor, Compound C, reversed the inhibitory effect of HS on microglial activation.

We also found that HS protected against acute injuries induced by HI, which confirmed our previous observations. Administration of the AMPK inhibitor, Compound C, prior to HS in immature mice significantly blunted the HS effects on edema (Fig. 2c, *p* < 0.01) and infarction (Fig. 2d, *p* < 0.05).

### HS suppressed HI-induced neuro-inflammation and promoted M2 microglia polarization in the lesioned cortex at 3 days post-HI

**Fig. 3:**
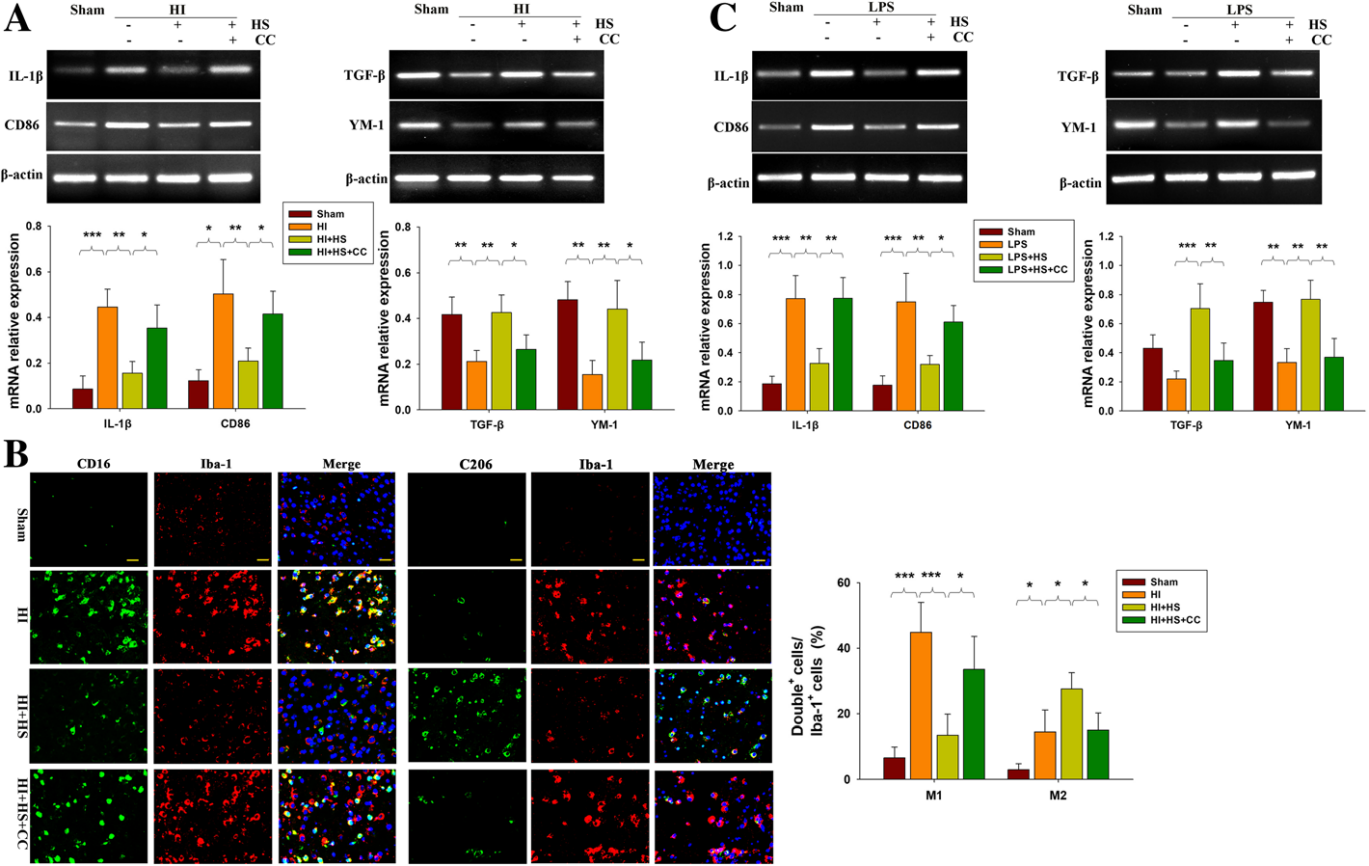
HS significantly alters expression profiles of inflammation mediators. **a** Representative RT-PCR photographs showing mRNA levels of IL-1β, CD86, TGFβ and YM-1 within ipsilateral cortex at 3 days following HI insult. Quantitative results of relative mRNA levels within each group. Values were normalized to β-actin. N = 4/group. **b** Representative photographs of double immunofluorescent staining of CD16 (green), CD206 (green), and Iba-1 (red) within the ipsilateral cortex at 3 days following HI insult. Scale bar = 20 μm. Six randomly captured images (× 20) were obtained for each section per animal. *N* = 4/group. **c** BV-2 cells treated with 1 μM HS with/without 500 ng/mL LPS or 1 μM Compound C for 2 h. The mRNA levels of IL-1β, CD86, TGFβ and YM-1 were determined with use of RT-PCR. N = 4/group. Values represent the mean ± SD, * *p* < 0.05, **p < 0.01, ***p < 0.001 according to ANOVA

HI insult induced classical M1 (IL-1β and CD86) and M2 (TGF- β and YM-1) responses in the lesioned cortex as compared with that observed in the Sham group (Fig. 3a, b). In response to HS treatment a lower expression of the pro-inflammatory factors IL-1β and CD86 (*p* < 0.01 for both) and higher expression of the anti-inflammatory mediators, TGF- β and YM-1 (*p* < 0.01 for both), were obtained (Fig. 3a).

As a means to further demonstrate that the effect of HS was exerted on microglial polarization, we used a co-staining approach involving CD16^+^/Iba1^+^ and CD206^+^/Iba1^+^ to identify the M1 and M2 phenotypes, respectively. The results of this assay revealed that both M1 and M2 phenotypes were significantly increased within the lesioned after HI. The number of M1 phenotypes (CD16^+^/Iba1^+^) in the cortex was significantly reduced (Fig. 3b, *p* < 0.001) and the number of M2 phenotypes (CD206^+^/Iba1^+^ cells) in the ipsilateral cortex significantly increased (Fig. 1b, *p* < 0.05) in the HI + HS group as compared with that in response to HI alone when determined at 3 days post-HI. Pre-treatment with an inhibitor of AMPK, Compound C, reversed this effect of HS on M2 microglial polarization (Fig. 3b).

In BV-2 cells, HS treatment decreased LPS-induced mRNA expression levels of IL-1β and CD-86 mRNA (*p* < 0.01 for both), while increasing TGF-β (*p* < 0.001) and YM-1 (*p* < 0.01) mRNA expressions; these effects were reversed by treatment with Compound C treatment (Fig. 3c).

### HS decreased NF-κB activation at 3 days following HI insult

**Fig. 4:**
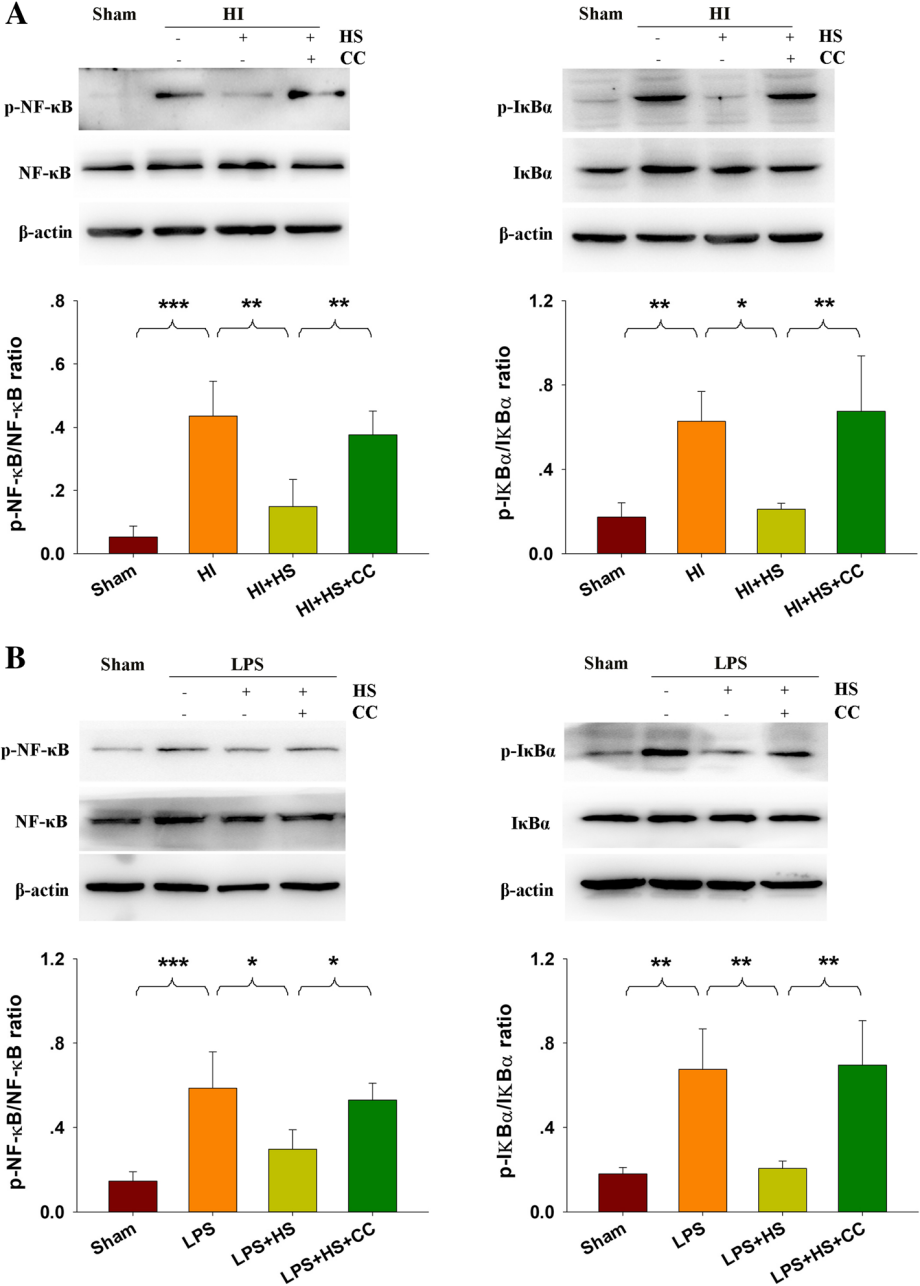
Effect of HS on NF-κB activation in lesioned cortex and BV-2 cells**. a** Representative immunoblots are shown for phosphorylated NF-κB (p-NF-κB), total NF-κB, phosphorylated-IκBα (p-IκBα), and total IκBα within ipsilateral cortex at 3 days following HI insult as determined with western blotting analysis. Quantification of the relative levels of p-NF-κB/NF-κB, p-IκBα/ IκBα within each group. *N* = 4/group. **b** BV-2 cells treated with 1 μM HS and with/without 500 ng/mL LPS for 2 h. The level of p-NF-κB/NF-κB, p-IκBα/ IκBα were measured by Western blot. *N* = 4/group. Values were normalized to β-actin. Values represent the mean ± SD, *p < 0.05, **p < 0.01, ***p < 0.001 according to ANOVA

Expressions of p-NF-κB and p-IκBa were increased in the HI group as compared with those of the Sham group at 3 days post-HI. HS treatment significantly decreased the expressions of p-NF-κB (*p* < 0.01) and p-IκBa (*p* < 0.05) as compared with that observed in the vehicle-treated HI group (Fig. 4a). In BV-2 cells, HS markedly downregulated the expressions of p-NF-κB (*p* < 0.05) and p-IκBa (*p* < 0.01) as compared with that observed following LPS treatment (Fig. 4b).

Results of the DHE assay revealed that HI insult increased ROS levels at 3 days after HI (*p* < 0.001, Additional file 4: Figure S3), which was attenuated by HS treatment (*p* < 0.001).

### Effects of HS on HS on synaptic loss in HI mice

**Fig. 5:**
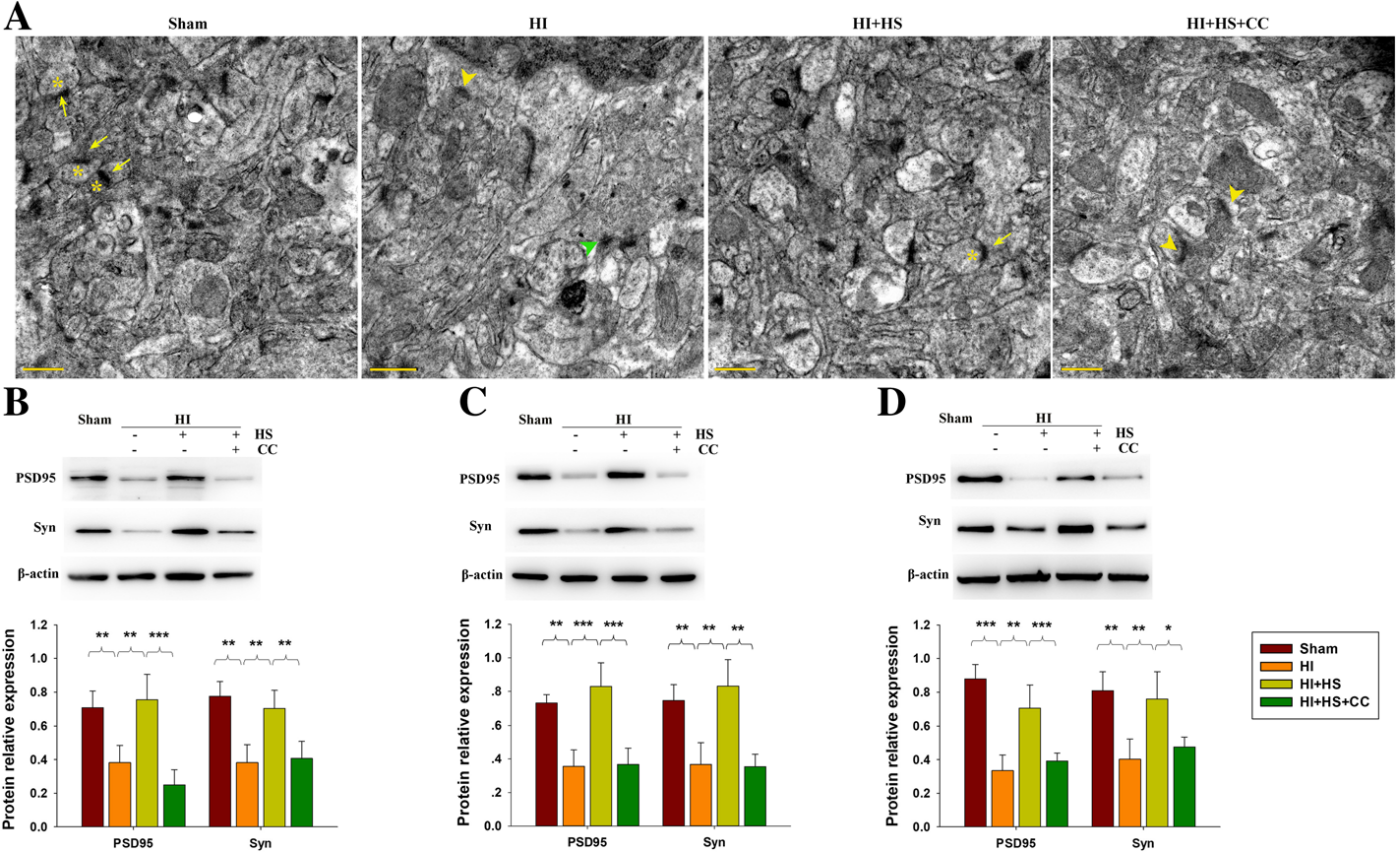
Effects of HS on structural remodeling of synapses following HI injury. **a** Representative electron micrographs at 28 days following HI indicating: * - presynaptic vesicles, Yellow arrow - postsynaptic partners, Green arrowhead - widened synaptic clefts, and Yellow arrowhead - destroyed synapses. Scale bar = 2 μm. **b-d** Levels of PSD95 and Syn within the ipsilateral cortex as examined at 3, 14 or 28 days post-HI with use of Western blot. Bar graphs show quantifications of protein levels at 3, 14 or 28 days. N = 4/group. Values represent the mean ± SD, * *p* < 0.05, **p < 0.01, ***p < 0.001 according to ANOVA

Based on electron microscopy images, we observed that synaptic clefts were widened and whole synapses destroyed by HI insult (Fig. 5a). In contrast, synaptic ultrastructure, number and densities within the HS-treated HI group were similar to that observed within the Sham group (Additional file 5: Figure S4 and Fig. 5a).

To assess synaptic modifications as induced by HS, we examined the expressions of Syn and PSD95, which are considered as markers of excitatory postsynaptic densities. In the ischemic cortex, expression levels of PSD95 and Syn were markedly downregulated at 3, 14, and 28 days after HI insult, while HS treatment significantly restored these HI-induced reductions in PSD95 (*p* < 0.01, *p* < 0.001, *p* < 0.01, respectively) and Syn (*p* < 0.01, *p* < 0.01, *p* < 0.01, respectively) expression (Fig. 5b–d). Compound C combined with HS significantly inhibited the effects of HS on Syn and PSD95 expression (Fig. 5).

### HS treatment rescues HI-induced synaptic loss linked to complement signaling

**Fig. 6:**
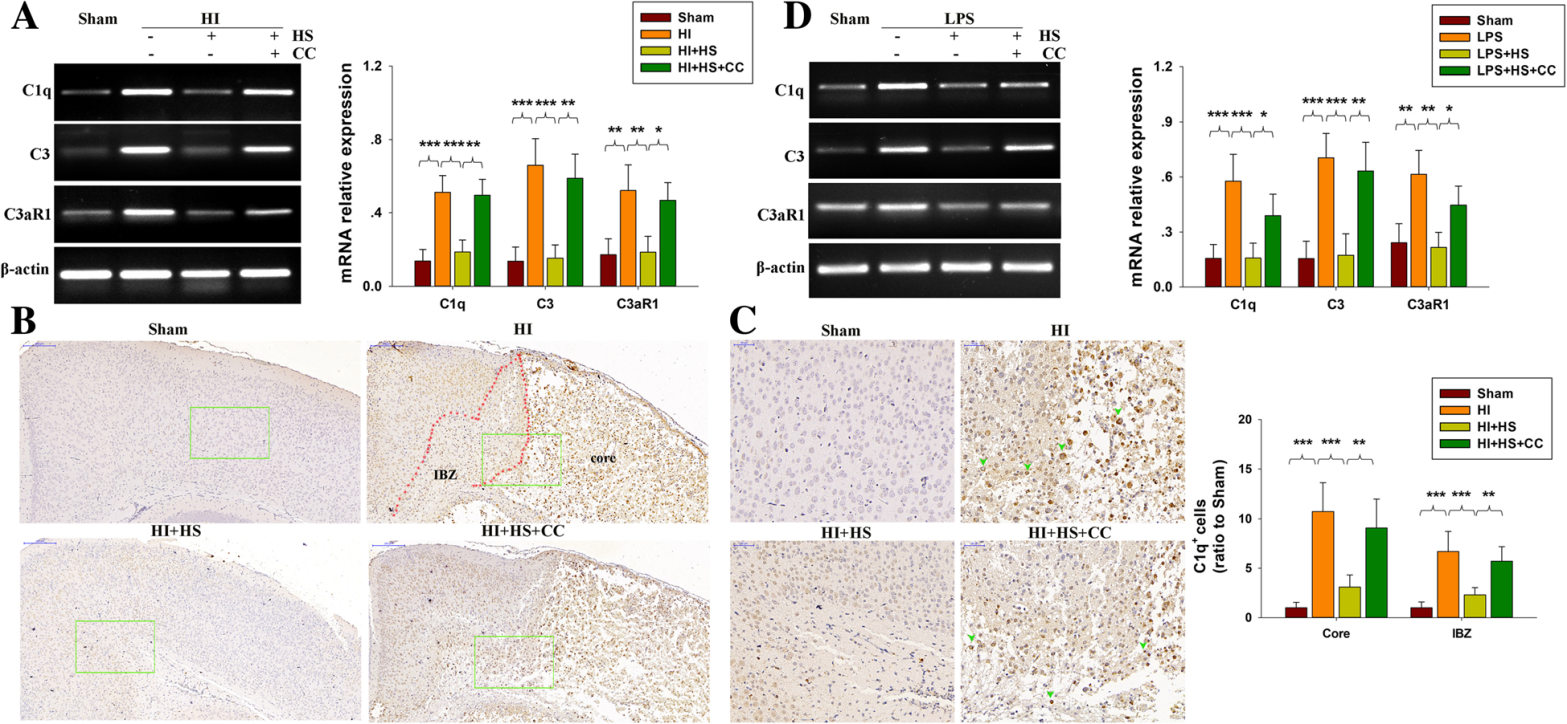
HS suppresses HI-induced complement activation in the lesioned cortex. **a** Representative RT-PCR photographs showing mRNA levels of C1q, C3 and C3aR1 within ipsilateral cortex at 3 days post-HI. Quantitative results of relative mRNA levels within each group. Values were normalized to β-actin. *N* = 4 mice/group. **b** Representative photographs of C1q staining within ipsilateral cortex taken at 72 h following HI. **c** Magnified views of boxed regions in B showing C1q staining. Quantification of C1q^+^ cells. *N* = 6/group. **d** BV-2 cells treated with 1 μM HS with/without 500 ng/mL LPS or 1 μM Compound C for 2 h. The mRNA levels of C1q, C3 and C3aR1 were determined with use of RT-PCR. Values represent the mean ± SD, *p < 0.05, **p < 0.01, ***p < 0.001 according to ANOVA

Complement activation participates in elimination of neuronal synapses in response to pathological conditions. Accordingly, we next examined whether HS treatment following HI insult was associated with inhibiting complement activation. While HI exposure increased the expressions of C1q, C3, and C3R1 at 3 days post-HI, HS significantly decreased the expression of C1q (*p* < 0.001), C3 (*p* < 0.001) and C3R1 (*p* < 0.01) as compared with that of the HI group (Fig. 6a). In BV-2 cells, HS treatment decreased LPS-induced C1q (*p* < 0.001), C3 (*p* < 0.001), and C3R1 (*p* < 0.01), these effects were reversed by treatment with Compound C treatment (Fig. 6b).

**Fig. 7:**
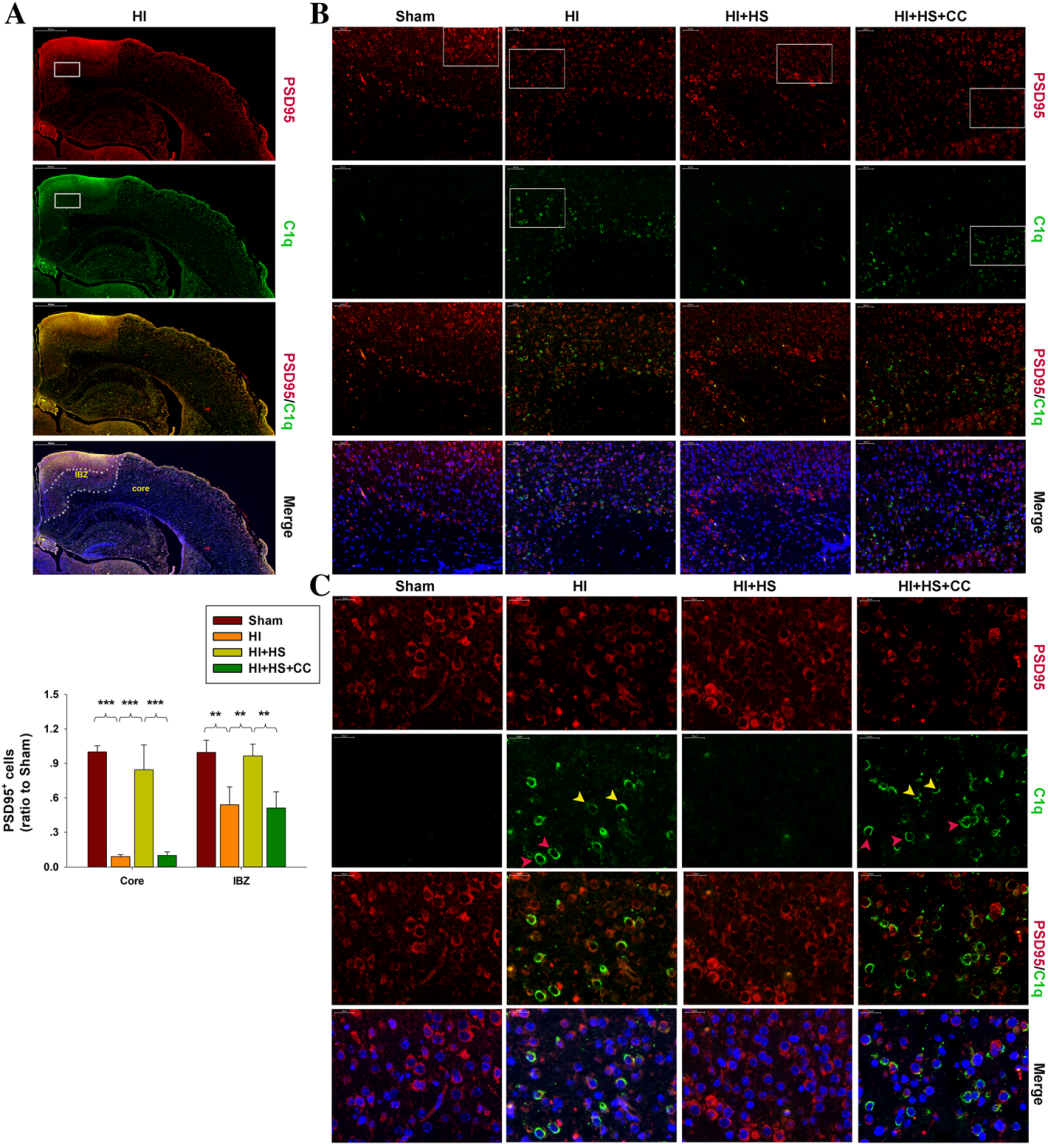
HS suppresses HI-induced synapse deficits linked to regulating complement expression in the lesioned cortex. **a** Double staining PSD95 (red) and C1q (Green) within the ipsilateral cortex were examined at 3 days post-HI. C1q labeled cells (green) were detected in the infract core and lesion boundary zone (IBZ). Scale bar = 500 μm. **b** Magnified views of boxed regions in A showing PSD95 and C1q staining. Scale bar = 50 μm. **c** Magnified views of boxed regions in **b** showing PSD95 and C1q staining. Yellow arrows showing PSD95 and C1q co-location. Red arrows showing C1q-positive cells and PSD95-negative cells. Scale bar = 20 μm. *N* = 4/group. Values represent the mean ± SD, **p < 0.01, ***p < 0.001 according to ANOVA

HI exposure produced a substantial increase in C1q expression within the infarct core region and lesion boundary zone, but not in the unaffected hemisphere as based on results from immunohistochemistry (Fig. 6c) and immunofluorescence (Fig. 7a–c) analyses. In contrast, HS treatment significantly inhibited this HI-induced increase in C1q levels within the infarct core region and lesion boundary zone (*p* < 0.001 for both, Fig. 6c).

With immunofluorescence staining, PSD95 positive cells were found to be conspicuous and uniform within the cortex of the Sham group. Following exposure to HI, PSD95 positive cells became patchy and less concentrated in the infarct core region (*p* < 0.001) and lesion boundary zone region (*p* < 0.01, Fig. 7a, b). However, HS treatment significantly restored PSD95 expression resulting from this HI insult within both regions (*p* < 0.001, *p* < 0.01, respectively, Fig. 7a).

Using PSD95/C1q double staining, we investigated the localization of PSD95 and C1q following HI insult. The result from this assay showed that HI exposure increased C1q immunoreactivity in the infarct core region and lesion boundary zone (Fig. 7b, c), while corresponding in location to the synaptic loss (indicated as decreasing PSD95 expression). HS treatment restored these changes in C1q and PSD95 expression resulting from HI insult (Fig. 7b, c).

### Effects of HS on neurological reflexes following HI insult

**Fig. 8:**
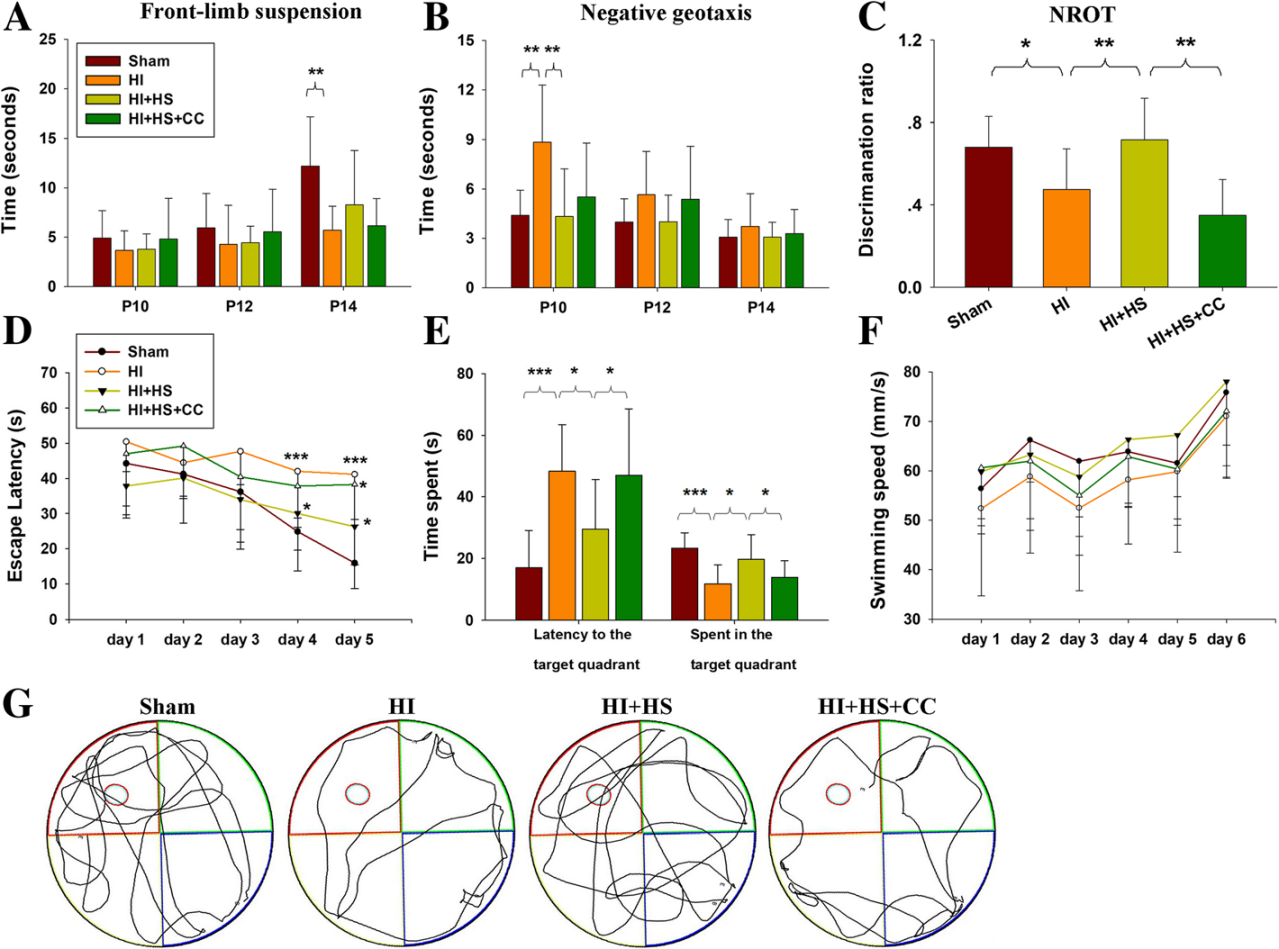
Effects of HS on neurological reflexes following HI insult. Daily performance of **a** front-limb suspension reflexes and **b** negative geotaxis of each group. **c** Comparisons of NORT test discrimination ratios which represent the ability to discriminate between a novel versus familiar object: novel/(novel + familiar time). **d** During each acquisition trial in the MWM test, escape latencies (in seconds) were measured and analyzed over days 1 to 5. **e** On the sixth day, the escape latency and percent time spent in the target quadrant were recorded (in seconds) and analyzed. **f** Swimming speeds of all groups from days 1 to 6 in the MWM test. **g** Display of tracks of all groups on day 6. Values represent the mean ± SD, *N* = 10/group. *p < 0.05, **p < 0.01, ***p < 0.001 according to ANOVA

The response latencies progressively decreased from P10 to P14 in each group in the front-limb suspension test [*F*(3,39) = 16.212, *p* < 0.001] and the negative geotaxis test [*F*(3,39) = 11.187, *p* < 0.001]. No statistically significant interactions were obtained between training days and groups in the front-limb suspension [*F*(3,39) = 2.124, *p* > 0.05] and negative geotaxis [*F*(3,39) = 1.747, *p* > 0.05] tests. At P14, HI animals exhibited shorter durations of suspension as compared to the Sham group ([*F*(3,36) = 4.946, *p* < 0.01]) and HS treatment had no effect on front-limb suspension durations in these mice (*p* > 0.05, Fig. 8a). Response latencies in the negative geotaxis test were significantly increased at P10 in the HI as compared with the Sham group ([*F*(3,36) = 5.379, *p* < 0.01]) and HS treatment significantly reversed these effects of HI on negative geotaxis responses (*p* < 0.01, Fig. 8b). Pre-treatment with Compound C significantly reversed the beneficial effects of HS on negative geotaxis.

In the NORT test, HI exposure resulted in a significant decline in discrimination ratios as compared with the Sham group (*F*[3, 36] = 9.195, *p* < 0.001; Fig. 8c). HS treatment resulted in a significant increase in discrimination ratios as compared with the HI group (*p* < 0.01; Fig. 8c), suggesting that HS treatment reversed HI-impaired object recognition memory. Pre-treatment with Compound C significantly reversed the beneficial effects of HS on discrimination ratios.

In MWM test, mean latencies progressively decreased over training days one to five in each group [*F*(3,36) = 13.745, *p* < 0.001] and no significant interaction between training days and groups was present [*F*(3,36) = 1.629, *p* > 0.05]. These data revealed that all mice showed the same improvements in spatial learning and memory over time, regardless of previous treatment. In addition, all the mice showed similar swim speeds during training, suggesting that any potential differences in spatial learning and memory could not be attributable to differences in swim speed (Fig. 8e).

In the initial 3 days of testing, animals in each group showed similar escape latencies in the visible-platform test (*p* > 0.05). However, mice of HI group showed significant increases in the escape latencies as compared with the Sham group on days 4 [*F*(3,36) = 4.136, *p* < 0.05] and 5 [*F*(3,36) = 10.233, *p* < 0.001] (Fig. 8d). In response to HS treatment, a progressive reduction in escape latencies was observed with differences being statistically significant on days 4 (*p* < 0.05) and 5 (*p* < 0.05), indicating learning improvement.

In the probe trial on day 6, the HI group spent an increased amount of time searching for the original platform [*F*(3,36) = 5.749, *p* < 0.01] and less time in the target quadrant in comparison to that of the Sham group [*F*(3,36) = 7.884, *p* < 0.001] (Fig. 8e), suggesting memory impairment. However, decreased escape latencies (*p* < 0.05) and increased amounts of time in the target zone (*p* < 0.05) were observed in the HS as compared with that of the HI group (Fig. 8e). Pre-treatment with Compound C significantly blocked these effects of HS upon MWM test results.

## Discussion

We have previously reported that HS can produce neuroprotection in HI treated neonatal mice. In this study, we expand upon these findings and examined whether HS would improve behavioral deficits and exert anti-inflammatory effects within HI mice. The main findings of our study include the following: (1) HS treatment attenuated HI-induced behavioral deficits, synapse loss and restored Syn and PSD95 expressions. (2) Beneficial effects of HS in neonatal mice were associated with an enhancement in microglia M2 polarization as achieved by promoting AMPK phosphorylation. (3) HS treatment inhibited complement-mediated synapse loss. (4) Suppression of NF-κB activation may contribute to the anti-inflammatory effects of HS.

### HS protected against HI-induced behavioral deficits through suppression of neuro-inflammation

Our present behavioral results reveal that treatment with HS post-HI injury prevented deficits in sensory and motor reflexes and cognitive functions in neonatal mice. In general, HS exerts a number of beneficial effects within the nervous system. For example, it has been reported previously that HS injection prevents cognitive deficits in animal models following traumatic brain injury, effects which were proposed to involve antioxidant activity and activation of BDNF-related synaptic plasticity [16]. HS has also been shown to alleviate peripheral nerve injury and promote functional recovery after sciatic nerve autografting in rats [17]. Moreover, hydrogen-rich water prevents the development and progression of depressive-like behavior in rats, through its capacity to inhibit inflammasome activation [18]. Based on these previously reported beneficial effects of HS on behavioral responses and different types of brain injuries, it would appear that HS could be used as a potential treatment against neurological disabilities following HI in neonatal mice.

As there is growing evidence suggesting that neurological disabilities are associated with increased neuro-inflammation, we examined alterations in inflammation after HI insult. It has been reported that the polarization of microglia from a M1 to M2 phenotype contributes to the progression of injury from HI [4, 19, 20]. As indicated above, M2-polarized microglia secrete anti-inflammatory cytokines and growth factors, thus protecting neurons from peripheral or central nervous system injuries. In the present study, we found that not only did HS treatment decrease the expression of pro-inflammatory mediators and increase the expression of anti-inflammatory factors in the lesioned cortex of HI mice, but also drove microglia to adapt a M2 phenotype. These beneficial effects were consistent with the behavioral responses obtained following HS treatment. Also in line with these in vivo results, HS inhibited LPS-induced pro-inflammatory factors and upregulated pro-inflammatory mediators in BV-2 cells. Similar to our current results are the findings from previous studies which indicate that HS exerts neuroprotective effects by attenuating neuro-inflammation. Moreover, treatment with hydrogen-enriched water reduces LPS-induced sickness and promotes recovery in mice, effects which are associated with downregulation of inflammatory gene expression and upregulation of anti-inflammatory gene expression [21]. Collectively, these reports combined with results from our current study suggest that the neuroprotective effects of HS are associated with a facilitation in the conversion of M1 to M2, leading to anti-inflammatory signaling and thus attenuating tissue damage after HI injury.

### HS promotes M2 microglia polarization by activating AMPK

AMPK is expressed in neurons, astrocytes, and microglia and plays various roles in promoting M2 polarization of macrophages/microglia [22, 23]. Synthetic, as well as some natural, compounds which suppress inflammation and promote a M1/M2 microglia shift by activating the AMPK pathway [24]. It has been reported that molecular H_2_ activates embryonic AMPK fibroblasts and neuronal cells and possesses anti-oxidant and anti-apoptotic properties [25, 26]. In this study, while AMPK activation was increased following HI insult or LPS stimulation, maximal AMPK responses were observed following HS treatment as demonstrated both *in vivo and in vitro*. Moreover, AMPK inhibitors block the effects of HS on neuro-inflammation and microglial polarization. Such findings suggest that the beneficial effects of HS against HI injury resulting from facilitation of the microglia shift to the M2 phenotype, occurs through the AMPK pathway. It is worth noting that although AMPK was activated following HI injury in neonatal mice, the beneficial or deleterious effects of this AMPK activation were dependent on whether it was activated prior to or during the injury. In specific, a prolongation of AMPK activation during HI injury will have the effect of aggravating cell death [27]. In future studies, we plan to investigate the long-term effects of HS treatment as related to AMPK activation.

### HS suppressed neuro-inflammation by inhibiting NF-κB activation

The NF-κB signaling pathway is essential for both innate and adaptive immunity. It has been reported that HS suppressed NF-κB activation, which then contributes to its beneficial effects upon inflammation [28]. Here, we found that HS treatment significantly inhibited HI/LPS-induced activation of NF-кB as demonstrated in vivo and in vitro. These effects were associated with a decrease in NF-κB-activated inflammatory genes and are in good agreement with previous studies. Several antioxidants suppress NF-κB activation through suppressing the generation of reactive oxygen intermediates [28, 29], which can either directly or indirectly promote the release of the inhibitory subunit IκB from NF-κB [30]. We propose that HS, acting as a novel antioxidant, blocks the activation of NF-kB in HI injury by inhibiting the production of reactive oxygen intermediates.

### HS restoration of synapse loss in the lesion boundary zone is linked to complement signaling

There is a considerable amount of literature documenting that synaptic structural remodeling, including changes in synapse morphology, number, and proteins, also results in changes in the number of dendritic spines [31]. In this study, the cognitive decline observed in HI exposed in neonatal mice was associated with synaptic deficits and a downregulation of synaptic protein expression. Consistent with our findings, results from previous studies have shown that synaptic density and synaptic proteins are reduced following HI insult in neonatal mice [32, 33]. Notably, the post-injury HS treatment, which improved neurological functions, was associated with significant increases in synaptic proteins, modulation of synapse morphology, and number. These findings are in accord with a previous report indicating that HS mediates synaptic plasticity by restoring levels of BDNF, synapsin I, and CREB in a traumatic brain injury animal model [16]. Accordingly, an additional mechanism through which HS treatment improves neurological functions appears to involve maintenance of synaptic integrity following HI injury.

The molecular mechanisms underlying synaptic remodeling in disease or injury are not known, but recent evidence has resulted in the suggestion that the complement cascade may play a role in this process. For example, C1q and the classical complement cascade are strongly activated in Alzheimer's disease and are associated with synapse loss [34]. The age-dependent increases in C1q are localized at or around synapses and play a role in the cognitive decline associated with normal brain aging [28]. However, C1q is only transiently expressed in developing retinas between P4 and P10 in synaptic regions, and C1q is rapidly upregulated within microglia and neurons following injury [35]. In this study, we found that the PSD95 labelling of cells was significantly reduced in the infract core and the lesion boundary zone, indicating synapse loss. Moreover, we also found that HI insult increased C1q, C3, and C3aR1 expression, and considerable amounts of C1q were deposited in the infract core and lesion boundary zone following injury. This C1q co-localized with damaged synapses, which suggests that elevated levels of C1q are directly linked to synapse loss following HI insult in the developing brain. Importantly, HS treatment post-injury significantly reduced this synaptic complement deposition and synapse loss. Moreover, Vicki et al. reported that exercise preserves neuronal function and survival in the retina via blocking complement-mediated synapse elimination [36]. Taken together, these data suggest that pharmacological inhibition of complement activation in HI mice may contribute to the restoration of synaptic deficits and neuronal damage and, in this way, serve as a neuroprotective therapy in HI.

Some potential limitations related to the present study include the mechanism involved with which C1q accumulates within close proximity to synapses in developing brains following HI. Moreover, the means through which the complement-dependent pathway impacts synapses remain obscure. Therefore, future studies will be required to address these issues.

## Conclusion

In summary, the present findings demonstrate that HS treatment prevented neuro-inflammation and behavioral dysfunction following HI insult. Some of the underlying mechanisms for these beneficial effects involve promotion of microglia M2 polarization and modulation of complement-mediated synaptic loss via activation of AMPK. Beneficial effect of HS on HI insult also appears to involve an inhibition of NF-κB activation.

## Additional files

Additional file 1: Reagent and PCR primers in the text. (ZIP 83 kb)

Additional file 2: **Figure S1.** Schema for HS and Compound C treatment schedule, behavioral experiments, and tissue preparation. (JPG 1255 kb)

Additional file 3: **Figure S2.** Effects of LPS on AMPK activation in microglia. (JPG 345 kb)

Additional file 4: **Figure S3.** Effects of HS on ROS levels in the lesioned cortex at 3 days post-HI. (JPG 1871 kb)

Additional file 5: **Figure S4.** Effects of HS on synapse number in the lesioned cortex at 28 days post-HI. (JPG 433 kb)

## Abbreviations

AMPK: AMP-activated protein kinase
CD86: Cluster of differentiation 86
CNS: Central nervous system
CREB: cAMP response element-binding protein
DAPI: 4′,6′-diamidino-2-phenylindole dihydrochloride hydrate
HI: Hypoxic-ischemic
HS: Hydrogen-rich saline
Iba-1: Ionized calcium-binding adapter molecule 1
IL: Interleukin
LPS: Lipopolysaccharide
NF-κB: Nuclear factor-kappa B
PFA: Paraformaldehyde
PSD-95: Postsynaptic density protein 95
ROS: Reactive oxygen species
RT-PCR: Reverse transcription–polymerase chain reaction
Syn: Synaptophysin
TGF-β: Transforming growth factor β
TNF-α: Tumor necrosis factor-α
TTC: 2,3,5-triphenyltetrazolium chloride monohydrate

## References

[1] Bryce J, Boschi-Pinto C, Shibuya K, Black RE, Group WHOCHER (2005). WHO estimates of the causes of death in children. Lancet.

[2] Hagberg H, Mallard C, Ferriero DM, Vannucci SJ, Levison SW, Vexler ZS, Gressens P (2015). The role of inflammation in perinatal brain injury. Nat Rev Neurol.

[3] Li B, Concepcion K, Meng X, Zhang L (2017). Brain-immune interactions in perinatal hypoxic-ischemic brain injury. Prog Neurobiol.

[4] Bonestroo HJ, Nijboer CH, van Velthoven CT, Kavelaars A, Hack CE, van Bel F, Heijnen CJ (2013). Cerebral and hepatic inflammatory response after neonatal hypoxia-ischemia in newborn rats. Dev Neurosci.

[5] Hu X, Li P, Guo Y, Wang H, Leak RK, Chen S, Gao Y, Chen J (2012). Microglia/macrophage polarization dynamics reveal novel mechanism of injury expansion after focal cerebral ischemia. Stroke.

[6] Jaworska J, Ziemka-Nalecz M, Sypecka J, Zalewska T (2017). The potential neuroprotective role of a histone deacetylase inhibitor, sodium butyrate, after neonatal hypoxia-ischemia. J Neuroinflammation.

[7] Ohsawa I, Ishikawa M, Takahashi K, Watanabe M, Nishimaki K, Yamagata K, Katsura K, Katayama Y, Asoh S, Ohta S (2007). Hydrogen acts as a therapeutic antioxidant by selectively reducing cytotoxic oxygen radicals. Nat Med.

[8] Ohta S (2012). Molecular hydrogen is a novel antioxidant to efficiently reduce oxidative stress with potential for the improvement of mitochondrial diseases. Biochim Biophys Acta.

[9] Saramago Eduardo A., Borges Gabriela S., Singolani-Jr Carlitos G., Nogueira Jonatas E., Soriano Renato N., Cárnio Evelin C., Branco Luiz G.S. (2019). Molecular hydrogen potentiates hypothermia and prevents hypotension and fever in LPS-induced systemic inflammation. Brain, Behavior, and Immunity.

[10] Hong Y, Chen S, Zhang JM (2010). Hydrogen as a selective antioxidant: a review of clinical and experimental studies. J Int Med Res.

[11] Cai J, Kang Z, Liu WW, Luo X, Qiang S, Zhang JH, Ohta S, Sun X, Xu W, Tao H, Li R (2008). Hydrogen therapy reduces apoptosis in neonatal hypoxia-ischemia rat model. Neurosci Lett.

[12] Cai J, Kang Z, Liu K, Liu W, Li R, Zhang JH, Luo X, Sun X (2009). Neuroprotective effects of hydrogen saline in neonatal hypoxia-ischemia rat model. Brain Res.

[13] Bai X, Liu S, Yuan L, Xie Y, Li T, Wang L, Wang X, Zhang T, Qin S, Song G (2016). Hydrogen-rich saline mediates neuroprotection through the regulation of endoplasmic reticulum stress and autophagy under hypoxia-ischemia neonatal brain injury in mice. Brain Res.

[14] McCullough LD, Zeng Z, Li H, Landree LE, McFadden J, Ronnett GV (2005). Pharmacological inhibition of AMP-activated protein kinase provides neuroprotection in stroke. J Biol Chem.

[15] Feather-Schussler DN, Ferguson TS. A battery of motor tests in a neonatal mouse model of cerebral palsy. J Vis Exp. 2016;117:53569 10.3791/53569 PMC5226120 27842358

[16] Hou Z, Luo W, Sun X, Hao S, Zhang Y, Xu F, Wang Z, Liu B (2012). Hydrogen-rich saline protects against oxidative damage and cognitive deficits after mild traumatic brain injury. Brain Res Bull.

[17] Zhang YG, Sheng QS, Wang ZJ, Lv LI, Zhao W, Chen JM, Xu H (2015). Hydrogen-rich saline promotes motor functional recovery following peripheral nerve autografting in rats. Exp Ther Med.

[18] Zhang Y, Su WJ, Chen Y, Wu TY, Gong H, Shen XL, Wang YX, Sun XJ, Jiang CL (2016). Effects of hydrogen-rich water on depressive-like behavior in mice. Sci Rep.

[19] Hellstrom Erkenstam N, Smith PL, Fleiss B, Nair S, Svedin P, Wang W, Bostrom M, Gressens P, Hagberg H, Brown KL (2016). Temporal characterization of microglia/macrophage phenotypes in a mouse model of neonatal hypoxic-ischemic brain injury. Front Cell Neurosci.

[20] Donega V, Nijboer CH, van Tilborg G, Dijkhuizen RM, Kavelaars A, Heijnen CJ (2014). Intranasally administered mesenchymal stem cells promote a regenerative niche for repair of neonatal ischemic brain injury. Exp Neurol.

[21] Spulber S, Edoff K, Hong L, Morisawa S, Shirahata S, Ceccatelli S (2012). Molecular hydrogen reduces LPS-induced neuroinflammation and promotes recovery from sickness behaviour in mice. PLoS One.

[22] Li C, Zhang C, Zhou H, Feng Y, Tang F, Hoi MPM, He C, Ma D, Zhao C, Lee SMY (2018). Inhibitory effects of betulinic acid on lps-induced neuroinflammation involve M2 microglial polarization via CaMKKbeta-dependent AMPK activation. Front Mol Neurosci.

[23] Zhou X, Cao Y, Ao G, Hu L, Liu H, Wu J, Wang X, Jin M, Zheng S, Zhen X (2014). CaMKKbeta-dependent activation of AMP-activated protein kinase is critical to suppressive effects of hydrogen sulfide on neuroinflammation. Antioxid Redox Signal.

[24] Xu Y, Xu Y, Wang Y, Wang Y, He L, Jiang Z, Huang Z, Liao H, Li J, Saavedra JM (2015). Telmisartan prevention of LPS-induced microglia activation involves M2 microglia polarization via CaMKKbeta-dependent AMPK activation. Brain Behav Immun.

[25] Lee J, Yang G, Kim YJ, Tran QH, Choe W, Kang I, Kim SS, Ha J (2017). Hydrogen-rich medium protects mouse embryonic fibroblasts from oxidative stress by activating LKB1-AMPK-FoxO1 signal pathway. Biochem Biophys Res Commun.

[26] Hoshino A, Costa-Silva B, Shen TL, Rodrigues G, Hashimoto A, Tesic Mark M, Molina H, Kohsaka S, Di Giannatale A, Ceder S (2015). Tumour exosome integrins determine organotropic metastasis. Nature.

[27] Rousset CI, Leiper FC, Kichev A, Gressens P, Carling D, Hagberg H, Thornton C (2015). A dual role for AMP-activated protein kinase (AMPK) during neonatal hypoxic-ischaemic brain injury in mice. J Neurochem.

[28] Tan YC, Xie F, Zhang HL, Zhu YL, Chen K, Tan HM, Hu BS, Yang JM, Tan JW (2014). Hydrogen-rich saline attenuates postoperative liver failure after major hepatectomy in rats. Clin Res Hepatol Gastroenterol.

[29] Schreck R, Meier B, Mannel DN, Droge W, Baeuerle PA (1992). Dithiocarbamates as potent inhibitors of nuclear factor kappa B activation in intact cells. J Exp Med.

[30] Schreck R, Rieber P, Baeuerle PA (1991). Reactive oxygen intermediates as apparently widely used messengers in the activation of the NF-kappa B transcription factor and HIV-1. EMBO J.

[31] Benson DL, Huntley GW (2012). Building and remodeling synapses. Hippocampus.

[32] Shao Guo, Wang Yongqiang, Guan Shenheng, Burlingame Alma L., Lu Fuxin, Knox Renatta, Ferriero Donna M., Jiang Xiangning (2017). Proteomic Analysis of Mouse Cortex Postsynaptic Density following Neonatal Brain Hypoxia-Ischemia. Developmental Neuroscience.

[33] Xin D, Chu X, Bai X, Ma W, Yuan H, Qiu J, Liu C, Li T, Zhou X, Chen W (2018). l-Cysteine suppresses hypoxia-ischemia injury in neonatal mice by reducing glial activation, promoting autophagic flux and mediating synaptic modification via H2S formation. Brain Behav Immun.

[34] Hong S, Beja-Glasser VF, Nfonoyim BM, Frouin A, Li S, Ramakrishnan S, Merry KM, Shi Q, Rosenthal A, Barres BA (2016). Complement and microglia mediate early synapse loss in Alzheimer mouse models. Science.

[35] Stevens B, Allen NJ, Vazquez LE, Howell GR, Christopherson KS, Nouri N, Micheva KD, Mehalow AK, Huberman AD, Stafford B (2007). The classical complement cascade mediates CNS synapse elimination. Cell.

[36] Chrysostomou Vicki, Galic Sandra, van Wijngaarden Peter, Trounce Ian A., Steinberg Gregory R., Crowston Jonathan G. (2016). Exercise reverses age-related vulnerability of the retina to injury by preventing complement-mediated synapse elimination via a BDNF-dependent pathway. Aging Cell.

