# Supplementary material for: Hydrogen-rich saline promotes microglia M2 polarization and complement-mediated synapse loss to restore behavioral deficits following hypoxia-ischemic in neonatal mice via AMPK activation
Source: J Neuroinflammation. 2019 May 18;16:104. doi: 10.1186/s12974-019-1488-2 (PMC6525972; doi:10.1186/s12974-019-1488-2)
Supplement: Supplementary file 1 — Reagent and PCR primers in the text. (ZIP 83 kb) [file 12974_2019_1488_MOESM1_ESM.zip › Supplemental Table 1 Reagent.pdf]

Supplemental Table 1 Reagent

Company	Description	Catalog number
Cell Signaling Technology, Inc. (Boston, MA, USA)	Rabbit monoclonal Phospho-NF-κB p65(Ser536) (93H1) antibody	#3033
Cell Signaling Technology, Inc. (Boston, MA, USA)	Rabbit monoclonal Phospho-AMPKα (Thr172) (40H9) antibody	#2535
Cell Signaling Technology, Inc. (Boston, MA, USA)	Rabbit monoclonal AMPKα(D5A2) antibody	#5831
Cell Signaling Technology, Inc. (Boston, MA, USA)	Rabbit monoclonal Phospho-p38 MAPK (Thr180/Tyr182) (3D7) antibody	#9215
Proteintech Group (Rosemont, IL, USA)	Rabbit Polyclonal p38 MAPK antibody	14064-1-AP
Cell Signaling Technology, Inc. (Boston, MA, USA)	Rabbit monoclonal Phospho- IkBα antibody (Ser32) (14D4)	#2859
Cell Signaling Technology, Inc. (Boston, MA, USA)	Rabbit monoclonal IκBα (44D4) antibody	#4812
GeneTex, Inc. (Irvine, CA,USA)	Mouse Monoclonal Iba-1 antibody	GTX632426
Abcam (Cambridge, MA, USA)	Mouse Monoclonal NeuN antibody [1B7] - Neuronal Marker	ab104224

Abcam (Cambridge, MA, USA)	Mouse Monoclonal C1q antibody	ab71089
Proteintech Group (Rosemont, IL, USA)	Rabbit Polyclonal P65 antibody	10745-1-AP
Zhongshan Golden Bridge Biotechnology (Beijing, China)	Mouse Monoclonal β-actin antibody	TA-09
Proteintech Group (Rosemont, IL, USA)	Rhodamine (TRITC) -conjugated goat anti-rabbit IgG	SA00007-2
Proteintech Group (Rosemont, IL, USA)	Fluorescein (FITC) -conjugated affinipure goat anti-mouse IgG	SA00003-1
Zhongshan Golden Bridge Biotechnology (Beijing, China)	Peroxidase-conjugated goat anti-rabbit IgG	ZB-2301
Zhongshan Golden Bridge Biotechnology (Beijing, China)	Peroxidase-conjugated goat mouse IgG	ZB-2305
Millipore Corporation (Billerica,MA, USA)	Enhanced chemiluminescence and PVDF membranes	No.IPVH00010

Roche Diagnostics Gmbh (Indianapolis, IN, USA)	PhosSTOP phosphatase inhibitor	P1082
Beyotime Institute of Biotechnology (Jiangsu, China)	RIPA	P0013B
Beyotime Institute of Biotechnology (Jiangsu, China)	PMSF	ST506-2
Beyotime Institute of Biotechnology (Jiangsu, China)	5× loading buffer	P0015L
TOYOBO (Tokyo, Japan)	ReverTra Ace Qpcr RT Kit	FSQ-101
CWBIO (Haimen, Jiangsu, China)	BCA protein assay kit	CW0014S
CWBIO (Haimen, Jiangsu, China)	TRIzon reagent	01761/20114-1
Solarbio Science & Technology Co (Beijing, China)	GolView	G8140
Millipore Corporation (Billerica,MA, USA)	Immobilon Western Chemiluminescent HRP Substrate	WBKLS0100
