# Supplementary figures and images for: Hydrogen-rich saline promotes microglia M2 polarization and complement-mediated synapse loss to restore behavioral deficits following hypoxia-ischemic in neonatal mice via AMPK activation
Source: J Neuroinflammation. 2019 May 18;16:104. doi: 10.1186/s12974-019-1488-2 (PMC6525972; doi:10.1186/s12974-019-1488-2)

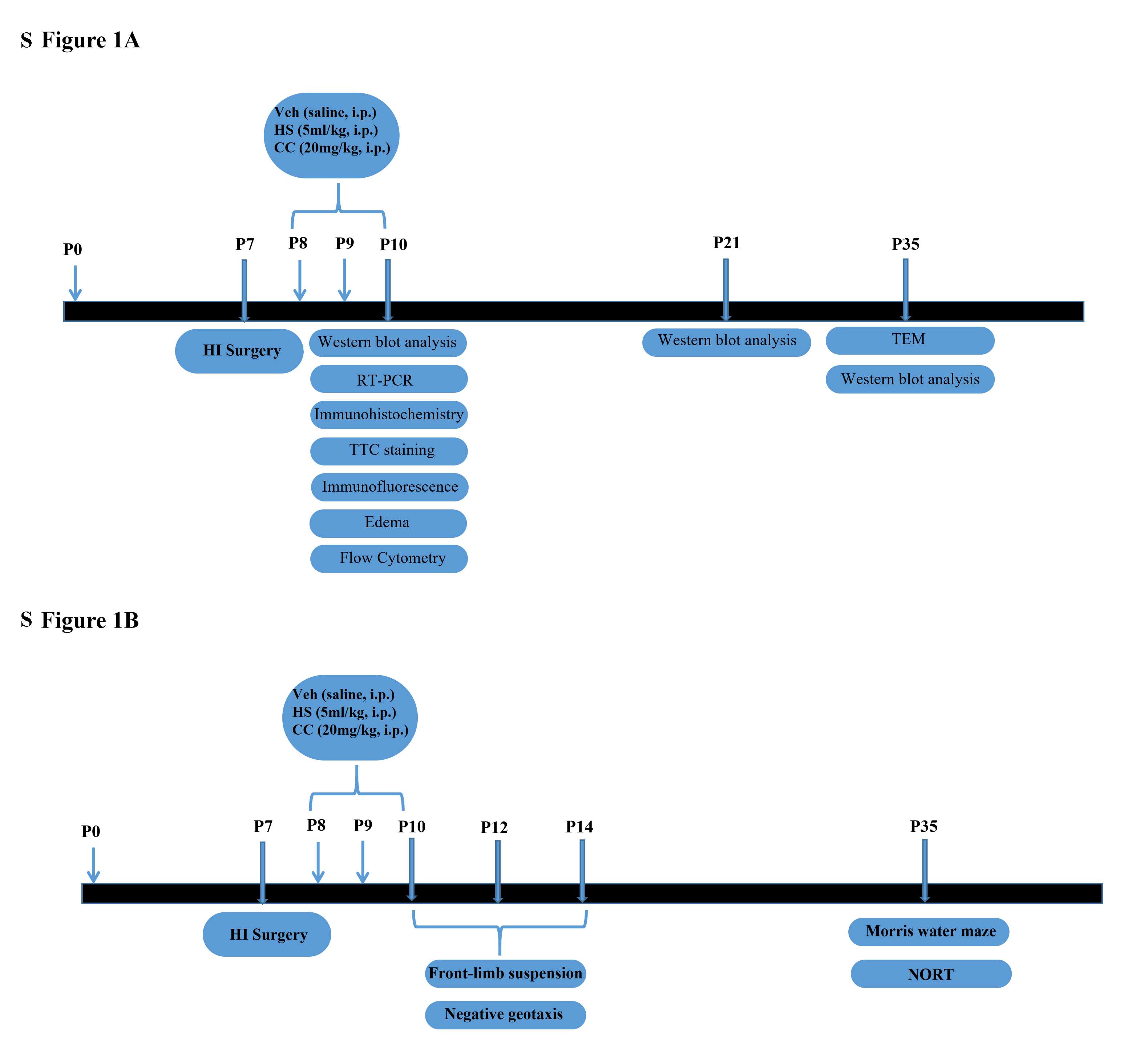

Supplement: Supplementary file 2 — Figure S1. Schema for HS and Compound C treatment schedule, behavioral experiments, and tissue preparation. (JPG 1255 kb) [file 12974_2019_1488_MOESM2_ESM.jpg]

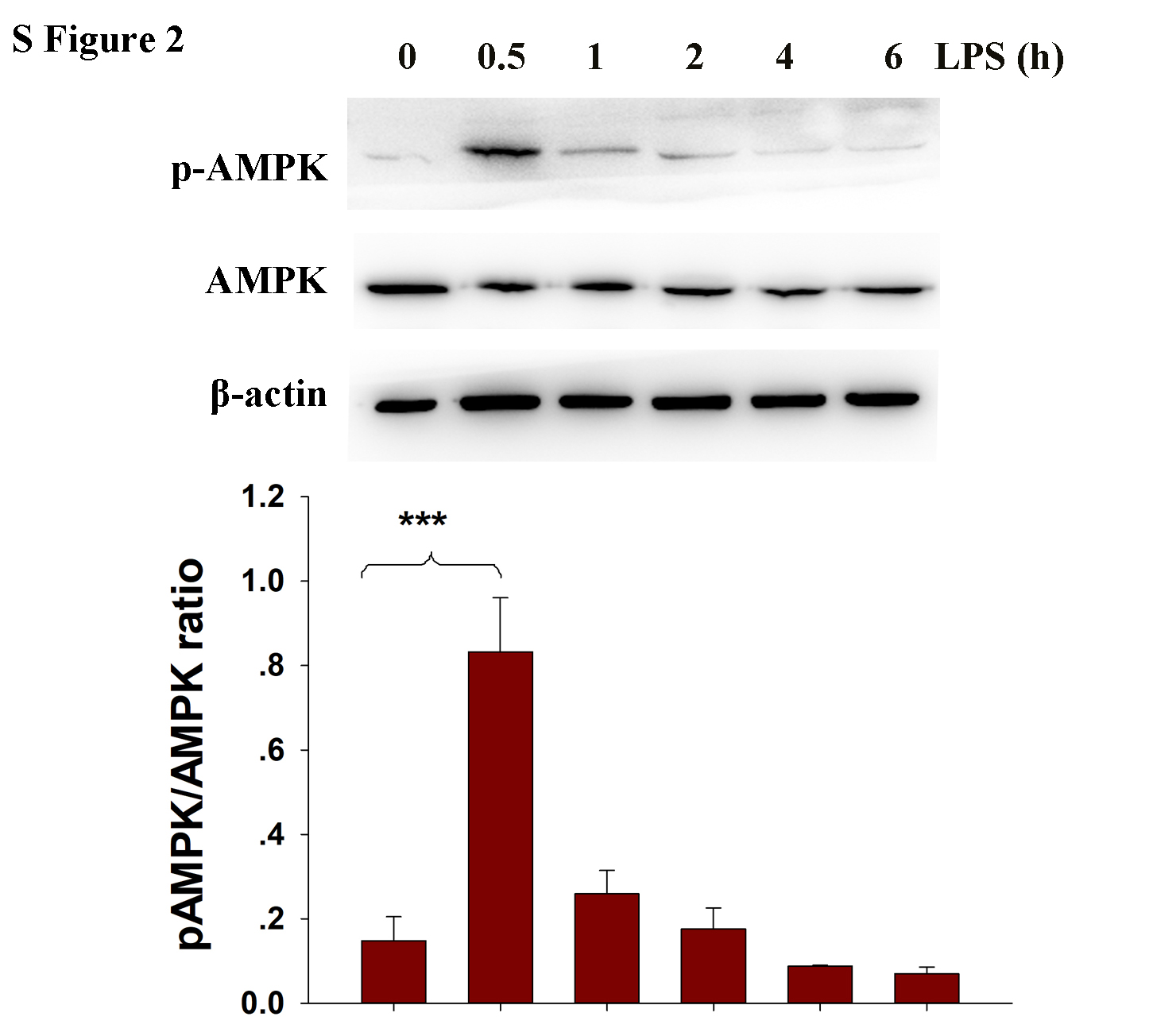

Supplement: Supplementary file 3 — Figure S2. Effects of LPS on AMPK activation in microglia. (JPG 345 kb) [file 12974_2019_1488_MOESM3_ESM.jpg]

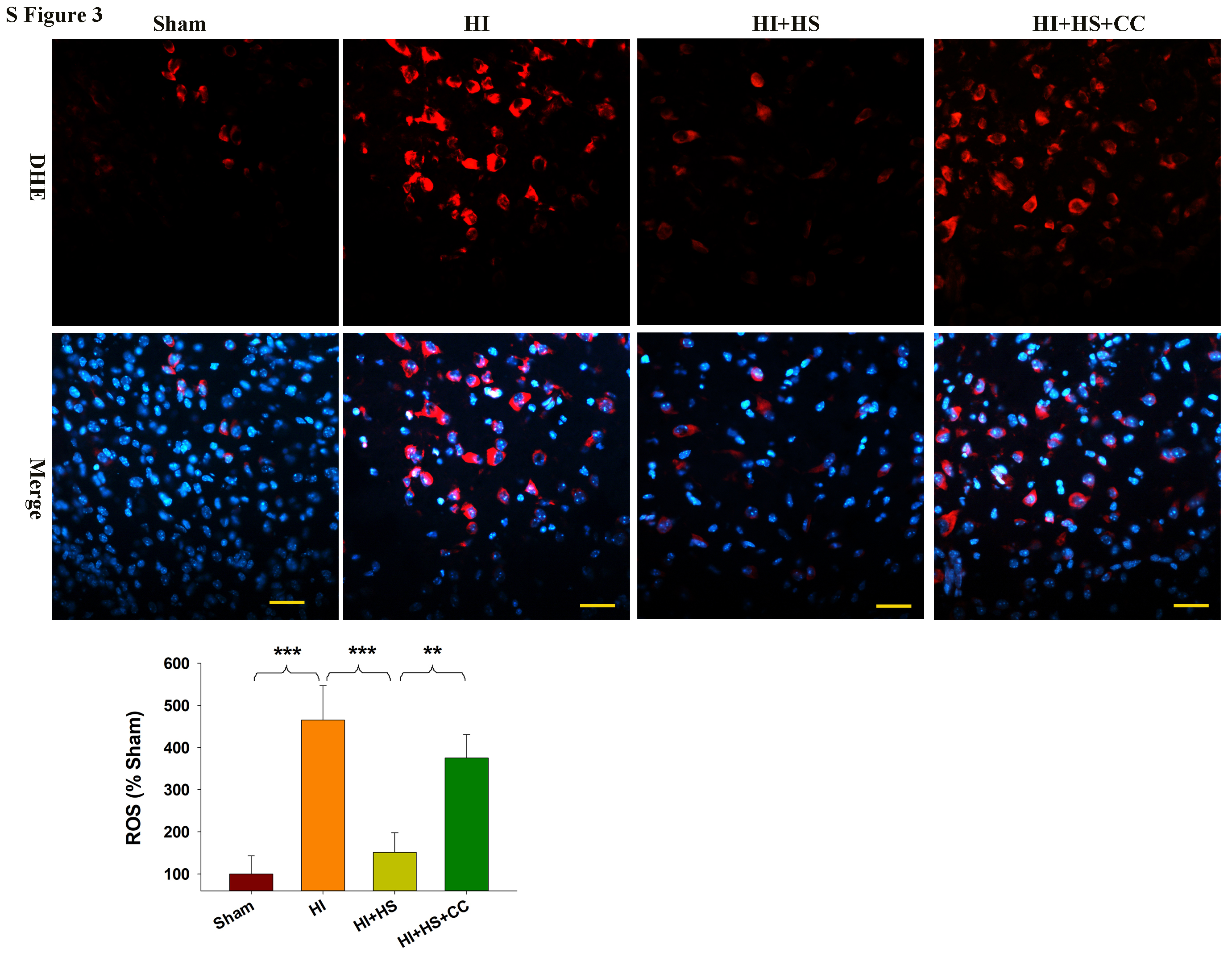

Supplement: Supplementary file 4 — Figure S3. Effects of HS on ROS levels in the lesioned cortex at 3 days post-HI. (JPG 1871 kb) [file 12974_2019_1488_MOESM4_ESM.jpg]

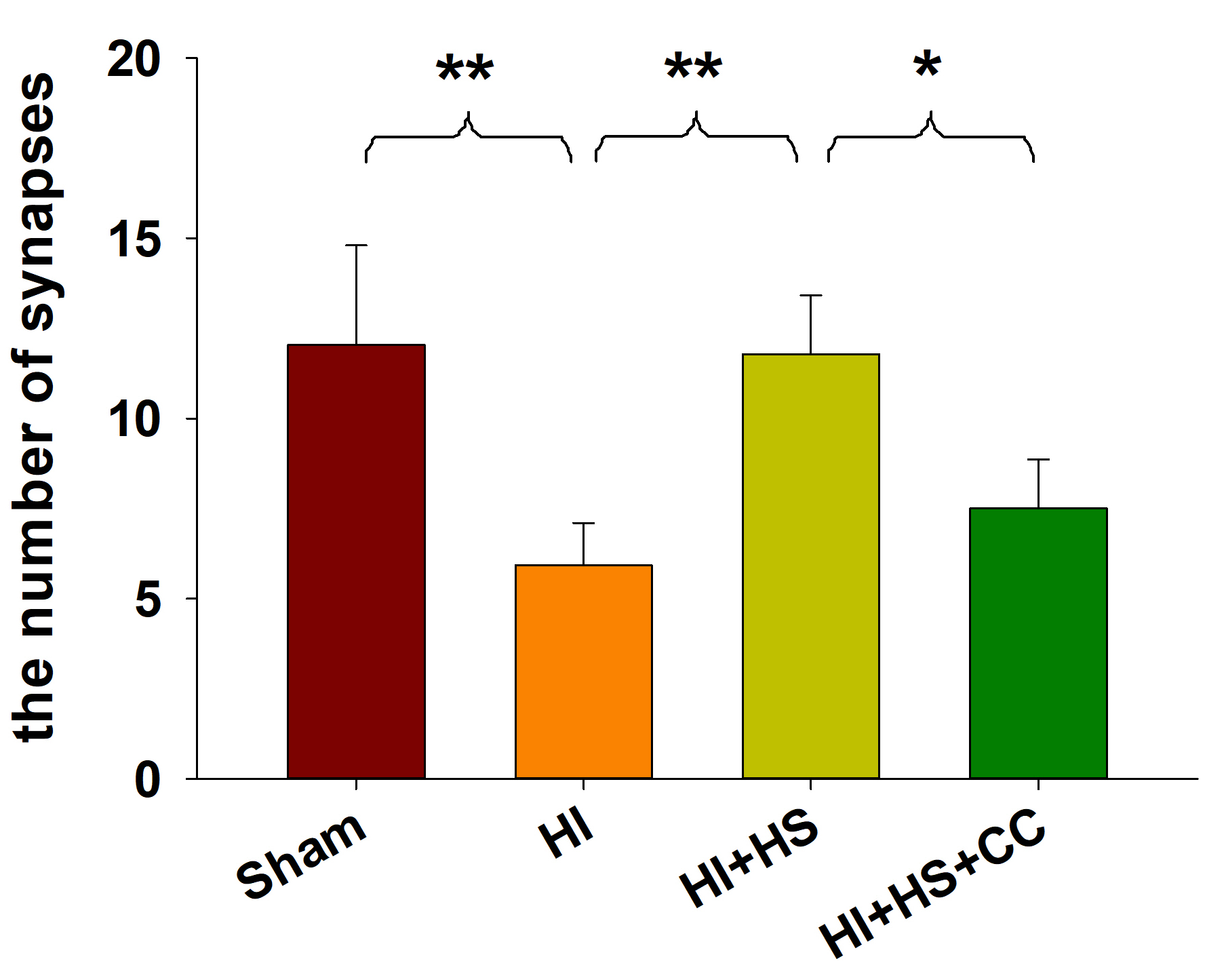

Supplement: Supplementary file 5 — Figure S4. Effects of HS on synapse number in the lesioned cortex at 28 days post-HI. (JPG 433 kb) [file 12974_2019_1488_MOESM5_ESM.jpg]
