# Supplementary material for: Hydrogen-rich saline promotes microglia M2 polarization and complement-mediated synapse loss to restore behavioral deficits following hypoxia-ischemic in neonatal mice via AMPK activation
Source: J Neuroinflammation. 2019 May 18;16:104. doi: 10.1186/s12974-019-1488-2 (PMC6525972; doi:10.1186/s12974-019-1488-2)
Supplement: Supplementary file 1 — Reagent and PCR primers in the text. (ZIP 83 kb) [file 12974_2019_1488_MOESM1_ESM.zip › Supplemental Table 2 PCR Primer.doc]

[**Table 1**](http://www.ncbi.nlm.nih.gov/pmc/articles/PMC2013981/table/tbl1/) PCR primers in the text

| *Gene* | *Forword (*5’→3’*)* | *Reverse (*5’→3’*)* |
| --- | --- | --- |
| TGF-β | GGT CCT TGC CCT CTA CAA CC | CCA CGT AGT AGA CGA TGC GC |
| Ym-1 | AGA CTT GCG TGA CTA TGA AGC ATT G | GCA GGT CCA AAC TTC CCA TCC TC |
| IL-1β | AAG ATG AAG GGC TGC TTC CAA ACC | ATA CTG CCT GCC TGA AGC TCT TGT |
| CD86 | TAA GCA AGG TCA CCC GAA AC | AGC AGC ATC ACA AGG AGG AG |
| C1q | AAT GAC GCT TGG CAA CGT GGT TAT C | ATG AGG AAT CCG CTG AAG ATG CTG |
| C3 | GCC TCT CCT CTG ACC TCT GG | AGT TCT TCG CAC TGT TTC TGG |
| C3aR1 | TGT TGG TGG CTC GCA GAT | GCA ATG TCT TGG GGT TGA AA |
| β -actin | CTA TTG GCA ACG AGC GGT TCC | CAG CAC TGT GTT GGC ATA GAG G |
